# Disease Tolerance and Pathogen Resistance Genes May Underlie *Trypanosoma cruzi* Persistence and Differential Progression to Chagas Disease Cardiomyopathy

**DOI:** 10.3389/fimmu.2018.02791

**Published:** 2018-12-03

**Authors:** Christophe Chevillard, João Paulo Silva Nunes, Amanda Farage Frade, Rafael Ribeiro Almeida, Ramendra Pati Pandey, Marilda Savóia Nascimento, Jorge Kalil, Edecio Cunha-Neto

**Affiliations:** ^1^INSERM UMR 1090, TAGC, Aix-Marseille Université, Marseille, France; ^2^Laboratorio de Imunologia, Instituto do Coracao, Hospital das Clinicas da Faculdade de Medicina da Universidade de Sao Paulo, São Paulo, Brazil; ^3^Disciplina de Imunologia Clínica e Alergia, Faculdade de Medicina da Universidade de Sao Paulo, São Paulo, Brazil; ^4^Institute for Investigation in Immunology (iii), INCT, São Paulo, Brazil; ^5^Department of Bioengineering, Brazil University, São Paulo, Brazil

**Keywords:** disease tolerance gene, pathogen resistance genes, *Tripanosoma cruzi*, chagas cardiomyopathy, interferon gamma

## Abstract

Chagas disease is caused by infection with the protozoan *Trypanosoma cruzi* and affects over 8 million people worldwide. In spite of a powerful innate and adaptive immune response in acute infection, the parasite evades eradication, leading to a chronic persistent infection with low parasitism. Chronically infected subjects display differential patterns of disease progression. While 30% develop chronic Chagas disease cardiomyopathy (CCC)—a severe inflammatory dilated cardiomyopathy—decades after infection, 60% of the patients remain disease-free, in the asymptomatic/indeterminate (ASY) form, and 10% develop gastrointestinal disease. Infection of genetically deficient mice provided a map of genes relevant for resistance to *T. cruzi* infection, leading to the identification of multiple genes linked to survival to infection. These include pathogen resistance genes (PRG) needed for intracellular parasite destruction, and genes involved in disease tolerance (protection against tissue damage and acute phase death—DTG). All identified DTGs were found to directly or indirectly inhibit IFN-γ production or Th1 differentiation. We hypothesize that the absolute need for DTG to control potentially lethal IFN-γ PRG activity leads to *T. cruzi* persistence and establishment of chronic infection. IFN-γ production is higher in CCC than ASY patients, and is the most highly expressed cytokine in CCC hearts. Key DTGs that downmodulate IFN-γ, like IL-10, and Ebi3/IL27p28, are higher in ASY patients. Polymorphisms in PRG and DTG are associated with differential disease progression. We thus hypothesize that ASY patients are disease tolerant, while an imbalance of DTG and IFN-γ PRG activity leads to the inflammatory heart damage of CCC.

## Introduction

Chagas disease is caused by infection with *Trypanosoma cruzi*, an obligatory intracellular parasite in the mammalian host. It is endemic in Latin America and affects over 8 million people worldwide, causing thousands of deaths each year. The pathogen naturally infects animals from central Argentina to the southern United States. Acute infection may lead to death in a small proportion of hosts, and survivors live with persistent infection with low parasitism. Chronically infected patients display differential progression. Nearly 30% of infected patients may develop life-threatening chronic heart disease due to an excessive inflammatory response, most others remaining in the asymptomatic/indeterminate (ASY) form with no heart disease, associated with a more immunomodulatory profile. Genes encoding resistance strategies that are shared against intracellular pathogens (1) have evolved for hundreds of millions of years, including the pathogen resistance receptors, TNF-α, and the IFN-γ-dependent immune response, among others (2). Most pathogen resistance genes (PRG) inhibit infection by directly reducing pathogen burden, and are related to immune-driven mechanisms—which, when in excess, can lead to death. Disease tolerance is an alternative strategy to avoid death after infection, whereby the pathogen's damaging effect on the host is mitigated. Disease tolerance is defined as the situation where an organism can bear a pathogen load without tissue damage and in the absence of a disease state. Disease tolerance genes (DTG)—which do not limit infection, but reduce its fitness costs—operate to minimize tissue damaging effects of the pathogen, leading to stress and damage reduction responses; DTG can also operate by counteracting excessive, tissue-damaging PRG activity (1, 3, 4). One such stress response pathways involved in disease tolerance is the oxidative stress response, whose master regulator is nuclear respiratory factor 2 (Nrf2) (3). By not limiting infection, the host remains a pathogen reservoir enabling transmission, in the absence of health consequences for itself, providing an evolutionary advantage for both host and pathogen. Disease tolerance is frequent in pathogens and hosts who have coevolved; while African monkeys which coevolved with the African virus Simian Immunodeficiency Virus (SIV) develop chronic infection with no disease, the same virus causes deadly disease in Asian macaques (5, 6). Evolutionary selection of DTG can be even more effective than that of PRG (4). A possible example of evolutionary selection of DTG against *T. cruzi* are the South American didelphid marsupials which coevolved with *T. cruzi* for over 40 million years and maintain high and long-lasting *T. cruzi* parasitemias in the absence of disease (7). We here hypothesize that the absolute need for DTG to control potentially lethal PRG activity against *T. cruzi* leads to parasite persistence and establishment of chronic infection. Our second hypothesis is that PRG and DTG also determine the differential progression of chronic Chagas disease toward tissue damage (CCC). According to this hypothesis, ASY patients are disease tolerant, while tissue damage in CCC is a consequence of insufficient DTG and/or excessive PRG activity. Along the review, we will provide evidence supporting both hypotheses.

## Pathogen resistance genes in *T. cruzi* infection

Most known pathogen resistance mechanisms against *T. cruzi* are immune-driven, directed at the intracellular forms of the parasite, and can be harmful if excessive. *T. cruzi* evades the powerful immune response and establishes a persistent infection with low parasitism. In order to obtain a list of known PRG and DTG, we surveyed the literature on *T. cruzi* infection of genetically deficient knockout mice. PRG were defined as genes essential for control of *T. cruzi* parasitism and needed for survival of infection; operationally, we identified as PRG those genes whose knockout led to increased pathogen load and mortality. DTG were defined as genes whose presence reduced mortality without any effect on *T. cruzi* control. We identified as DTG those genes whose knockout led to reduced parasitism and increased mortality. Table 1 lists the PRG and DTG identified in our literature review. Most PRG belong to the *TLR-MYD88-IL12-IFNG* pathway, *IL17* pathway, cell migration, inflammasome and other pathways involved in restriction of intracellular pathogen growth. Mice genetically deficient on *TLR4, TLR7*, and *TLR9, MYD88*, and *UNC93B1* display increased blood parasitism and mortality (8–13). Likewise, mice genetically deficient of *IL12A, IL12B*, and *STAT4*, essential for the differentiation of IFN-γ-producing Th1 cells, also display intense tissue and blood parasitism with increased mortality (15, 33). Along with TLR genes, *IFNG* is one of the main PRG involved in *T. cruzi* parasite control (43). Mice genetically deficient on *IFNG* or *STAT1* display drastically augmented *T. cruzi* parasitism and 100% mortality 13 days after infection (20, 21, 24). It was shown that *T. cruzi* amastigotes themselves dephosphorylate STAT1 serine residues, inhibiting IFN-γ signaling; evasion of IFN-γ signaling is further proof of the importance of the IFN-γ in the control of intracellular parasitism (44). IFN-γ-dependent PRG, like *TNFA* and *NOS2*, play a major role in resistance to *T. cruzi* (45, 46). The key role of TNF-α in *T. cruzi* control has been shown in TNFA-receptor 1 knockout mice (*TNFRSFA1*^−/−^), which display an increased number of blood and tissue parasites and shortened survival time (26). Platelet-activating factor (*PAF*) KO mice are more susceptible to *T. cruzi* infection than wildtype mice, and its protective effects depend on TNF-α-dependent NO production. In the context of protection against *T. cruzi*, IFN-γ, and TNF-α synergistically induce NF-kB activation to control *T. cruzi* parasitism and mortality in mice, by upregulating the expression of the PRG inducible nitric oxide synthase (*NOS2*), leading to the production of large amounts of NO and microbicidal reactive nitrogen species (RNS) (45, 46). *NOS2*-KO mice are susceptible to *T. cruzi* infection, with increased parasite burden and mortality due to lack of NO production (15, 47). Interestingly, constitutive *NOS1*-KO mice also showed increased parasitism and mortality (27). Genetic deficiency of macrophage *PI3KCG* increases susceptibility to *T. cruzi* infection; *PI3KCG* expression correlates with *IFNG* expression in CCC myocardium (38). IFN-γ increases ROS generation through induction of NADP oxidases (NOX2) and mitochondrial ROS via NF-kB activation (47, 48). Mice knockout for *NOX2* displayed increased *T. cruzi* tissue parasitism and mortality due to the lack of type 1 cytotoxic T cells (29). IFN-γ-induced ROS enhances

**Table 1:** *T. cruzi* pathogen resistance and disease tolerance genes.

**Symbol**	**Name**
**PATHOGEN RESISTANCE GENES**	
*tlr4* (8) *tlr7* (9) *tlr9* (9, 10)	Toll-like receptor 4 Toll-like receptor7 Toll-like receptor 9
*unc93b1* (9)	Unc-93 homolog B1, TLR signaling regulator
*myd88* (9, 11–13)	Myeloid differentiation primary response 88
*il6* (14) *il12* (15) *il12a* (16) *il12b* (17) *il17a* (18, 19)	Interleukin 6 Interleukin 12A Interleukin 12B Interleukin 17A
*ifng* (13, 15, 20–23)	Interferon-γ
*stat1* (24) *stat4* (25)	Signal transducer and activator of transcription 1 Signal transducer and activator of transcription 4
*tnfrsf1a* (26)	TNF receptor superfamily member 1A
*nos1* (27)	nitric oxide synthase 1
*nos2* (15, 22)	nitric oxide synthase 2, inducible
*casp1* (28)	caspase 1
*pycard* (28)	Asc/PYD and CARD domain containing
*ncf1* (29)	P47phox/neutrophil cytosolic factor 1
*ccl2* (30)	C-C motif chemokine ligand 2
*ccr5* (31, 32)	C-C motif chemokine receptor 5
*icam1* (33)	intercellular adhesion molecule 1
*cd28* (34)	CD28 antigen
*irgm1* (23)	immunity-related GTPase family M member 1
*ptafr* (35)	platelet-activating factor receptor
*lgals1* (36)	Galectin-1/lectin, galactose binding, soluble 1
*pnpla8* (37)	Phospholipase A2γ (iPLA2γ)/patatin-like phospholipase domain containing 8
*pi3kcg* (38)	phosphatidylinositol-4,5-bisphosphate 3-kinase catalytic subunit gamma
**DISEASE TOLERANCE GENES**	
*il6* (39)	Interleukin 6
*il10* (40, 41)	Interleukin 10
*il23* (39)	Interleukin 23
*il17ra* (42, 39)	Interleukin 17 receptor A
*ebi3* (39)	Epstein-Barr virus induced gene 3/ Interleukin-27p28

peroxynitrite anion (ONOO^−^) production, a strong oxidant arising from the reaction of NO with superoxide radical (O${}_{2}^{-}$) (49). ONOO^−^ induces damage to multiple molecules and is one of the ultimate effectors of parasite killing. Peroxynitrite promotes morphological disruption of internalized parasites, and induces severe alterations of energy metabolism, calcium homeostasis, and trypanothione depletion, severely impairing parasite redox homeostasis (50, 51). In addition, IFN-γ can also exert its protective effects *in vivo* in a NO-independent manner, through complementary events of protection against *T. cruzi*, such as production of other macrophage-derived effector molecules, MHC class II induction, CD4+ T cell polarization, IgG isotype switch to IgG2a and TLR induction (52–54). *IRGM1*, an IFN-γ-dependent GTPase, plays a key role in phagosome maturation and in the killing of intracellular pathogens contained in vacuoles (55).

**Figure 1:**
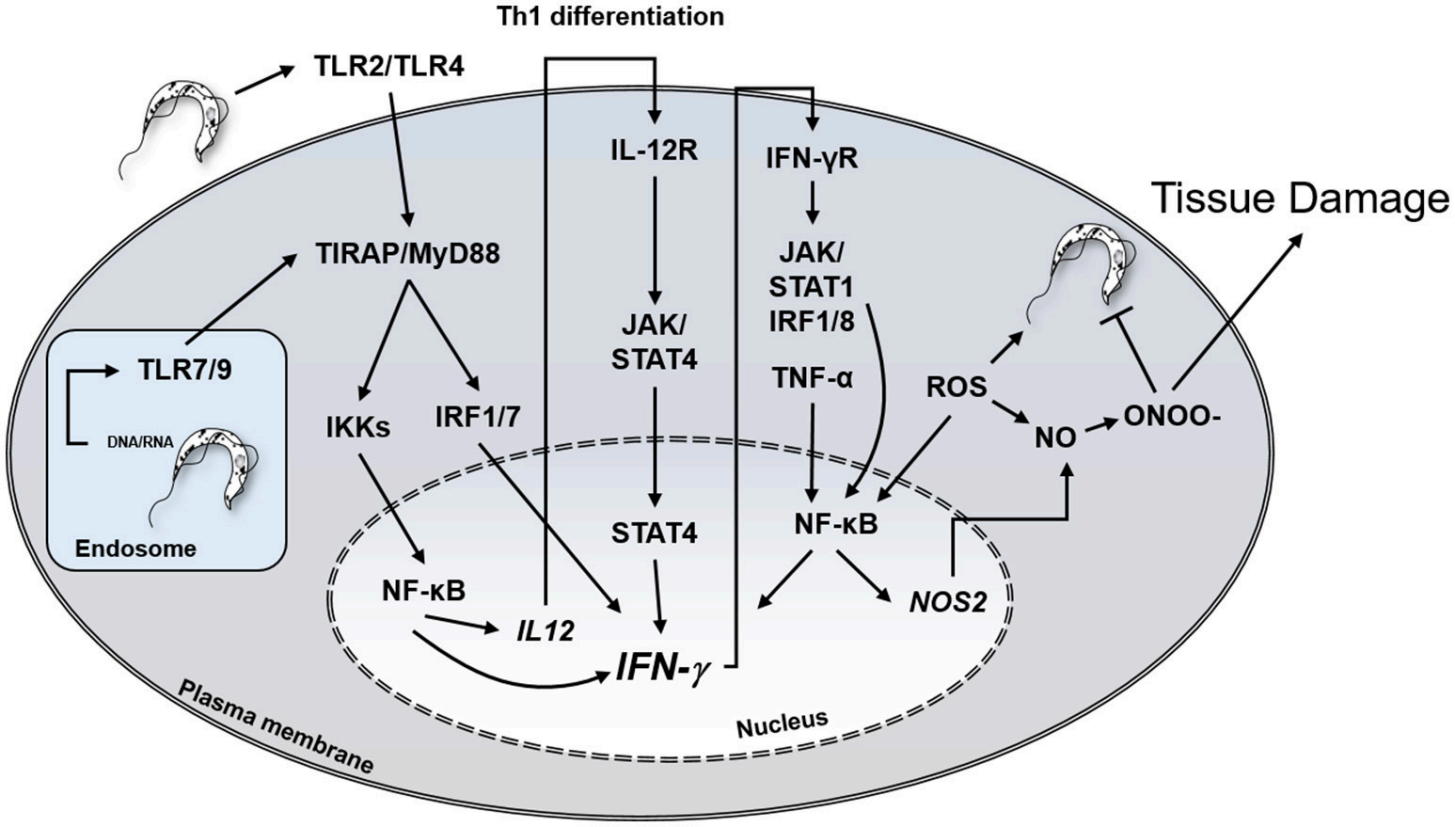
Central role of IFN-γ in protection against *T. cruzi* and potential host cell damage by peroxynitrite. IFN-γ, interferon-gamma; TLR, Toll-like receptor, IL12R, IL-12 receptor; IFNγR, IFN-γ receptor; JAK, Janus Associated Kinase; NO, nitric oxide; ONOO-, peroxynitrite; IRF, Interferon regulatory factor; STAT, Signal Transducer And Activator Of Transcription; ROS, reactive oxygen species.

In addition to inflammatory cells, several other cell types, including cardiomyocytes, fibroblasts and astrocytes, bear IFN-γ receptors and respond to the cytokine (56, 57). IFN-γ activates *T. cruzi*-infected macrophages and cardiomyocytes to produce TNF-α, NO, microbicidal ROS, and RNS. *In vitro* treatment with IFN-γ/TNF-α and IL1-β of mouse cardiomyocytes infected with *T. cruzi* resulted in NO production and elevated trypanocidal activity (58, 59). The primary role of IFN-γ mediating protection against intracellular *T. cruzi* infection is depicted in Figure 1.

*IL17A* is also a key PRG, and Th17 T cells may confer stronger protection against *T. cruzi*-related mortality than Th1 cells (60). IL-17A signaling is mainly dependent on TNF-α receptor associated factor 6 (TRAF6), but it can strongly promote TNF-α-induced NF-kB signaling by stabilizing pro-inflammatory mRNAs (61). Indeed, *IL17A* KO mice displayed an increase in blood and tissue parasitism with increased mortality. This was related to an impairment of leukocyte migration and activation of immune cells in the sites of parasite infection, as well as by reduced production of NO (18, 19). IL-17A also increases the persistence time of *T. cruzi* in the parasitophorous vacuole, enhancing exposure time of *T. cruzi* to the antimicrobial environment of endolysosomes, which can be further enhanced by IFN-γ-induced mechanisms (18). Genetic depletion of key inflammasome-related genes *CASP1* and *PYCARD*/Asc induce enhanced mortality and increased parasitism (28). Mice knockout for genes involved in migration pathways, like chemokines/receptors *CCL2, CCR5*, and adhesion molecule *ICAM1* also develop increased parasitism and decreased survival, in line with the impaired recruitment of leukocytes to sites of parasite replication (30–33). *CD28* KO mice display T cell activation defects, and *NCF1* (p47phox/NOX2) display a reduced type 1 CD8+ T cell response, leading to impaired control of parasitism and increased susceptibility to infection (29, 34). Although *IL27RA* KO mice develop increased parasitism and mortality and was a priori classified as a PRG, these mice develop grossly increased IFN-γ and inflammatory cytokine production, and death was related to the IFN-γ dependent tissue inflammation, a phenotype similar to that of *IL10* KO mice (62). This is in line with the known effect of IL27RA signaling in the control of IFN-γ and inflammatory cytokines. Phospholipase A2 γ knockout mice show increased susceptibility to *T. cruzi* infection, and decreased arachidonic acid and prostaglandin E2 production; the mechanism of protection is still obscure (37). Galectin-1 KO mice displayed increased parasitism and mortality upon infection with the Tulahuen strain, classifying it as a PRG (36). However, subsequent studies with infection of Galectin-1 with the RA strain showed reduced parasitism and mortality, which is not a PRG profile (63). This suggests the phenotypes may change according to the *T. cruzi* strain being tested and site of infection.

## Disease tolerance genes in *T. cruzi* infection

Table 1 shows the *T. cruzi* DTG. Remarkably, all *T. cruzi* DTG (*IL10, Ebi*-IL27p28*, IL17RA, IL23, IL6*) shared as a common feature the ability to reduce IFN-γ production or Th1 differentiation. In addition to decreased parasitism and increased mortality, inflammation was upregulated in all DTG-deficient mice. Infection of IL-10 deficient mice is accompanied by increased release of IFN-γ, TNF-α, IL-12, and RNS (40). Mechanistically, IL-10 is a potent inhibitor of monocyte-macrophage activation and NK cell activity and can inhibit the synthesis of TNF-α and IL-12 and IFN-γ (64, 65). Mice genetically deficient of IL-17RA or IL-23 showed increased mortality due to a shift to a Th1 profile after infection and augmented IFN-γ and TNF-α levels in the heart (39, 42, 66). IL17RA signaling downregulated T-bet expression, and reduced Th1 T cell differentiation, and further downregulated IFN-γ production in acute infection by recruiting IL-10-producing suppressive neutrophils (42). Paradoxically, IL-17A is a PRG (18). It is possible that a engagement of the IL-17 receptor by a different IL-17 family member can be responsible for the DTG effect of *IL17RA*. IL-23 negatively regulates IL-12-induced IFN-γ production in CD8+ T cells by reducing STAT4 phosphorylation, independently of IL-17A, IL-17F, or IL-22 (18, 25, 67, 68). In addition, IL-23 is a key stimulatory cytokine for Th17 and innate “type 17” cells that can respond immediately to pathogenic insults; IL-23 may thus also suppress IFN-γ by promoting IL17RA signaling (69). Infection of *Ebi3*/IL-27p28 deficient mice is accompanied by increased IFN-γ production, with augmented Th1 immune response (70). Mechanistically, Ebi3 signaling modulates overproduction of IFN-γ, by inducing a population of IL-10 producing Tr1 T cells (39). On the other hand, IL-6 can be both a PRG and a DTG, depending on the model of *T. cruzi* infection. IL-6's DTG activity may be secondary to inhibition of Th1 differentiation through enhancing IL-4 production in CD4+ T cells (71), while its PRG activity may be explained by its ability to upregulate endothelial adhesion molecules, facilitating lymphocyte migration into non-lymphoid tissues (14, 71). We will discuss evidence that *IFNG*, a major PRG that operates as a key player in pathogen protection and is the culprit of tissue damage in Chagas disease, is the main target of modulation by DTGs with relevance in both the acute infection and differential progression of chronic disease.

## Immune dynamics in acute *T. cruzi* infection

*T. cruzi* subverts a highly conserved cellular pathway for the repair of plasma membrane lesions and explores endogenous cellular machinery for invasion, escape from the parasitophorous vacuole, which allows intracytoplasmic survival, and replication (72). The intracellular life cycle of *T. cruzi* is a major target of the antiparasite response (73). Extracellular *T. cruzi* components, such as trypomastigote-derived glycosylphosphatidylinositol (tGPI) and glycoinositolphospholipid (eGIPL) engage membrane Toll-like receptors (TLR) 2 and 4 (74). *Trypanosoma cruzi* is internalized by several different mechanisms, but end up in the phagolysosomal compartment, where *T. cruzi* DNA and RNA engage TLR7 and TLR9. TLR engagement promotes the Myd88-mediated activation of NF-kB. Lysosome acidification promotes escape of *T. cruzi* to the cytoplasm, where it differentiates into the replicative amastigote forms. Amastigote replication in the cytoplasm leads to activation of inflammasomes that can induce inflammatory cytokines and NF-kB activation. This induces pro-inflammatory cytokines including IL-12, a PRG which elicits differentiation of IFN-γ-producing Th1 cells soon after infection, promoting Th1 cell differentiation. IFN-γ induces expression of multiple other PRGs, such as *TNFA* and *NOS2* (45, 46). Recent studies have reported that *IL17A* is a key PRG, plays a major protective role during the acute phase of infection (60). A strong antibody response is also triggered, but apparently has a lower effect on parasitism than innate immunity and acquired IFN-γ-dependent CD4+ and CD8+ T cell responses (53).

Although powerful, the immune response that occurs during acute infection leads to partial parasite control. *T. cruzi* evades complete eradication, leading to the establishment of a chronic persistent infection with low parasitism. *T. cruzi*-infected individuals maintain increased production of inflammatory/Th1 cytokines like IFN-γ and TNF-α as compared to healthy individuals, as a result of persistent stimulus of innate and specific immunity (75).

## Immune dynamics and differential disease progression to CCC

**Figure 2:**
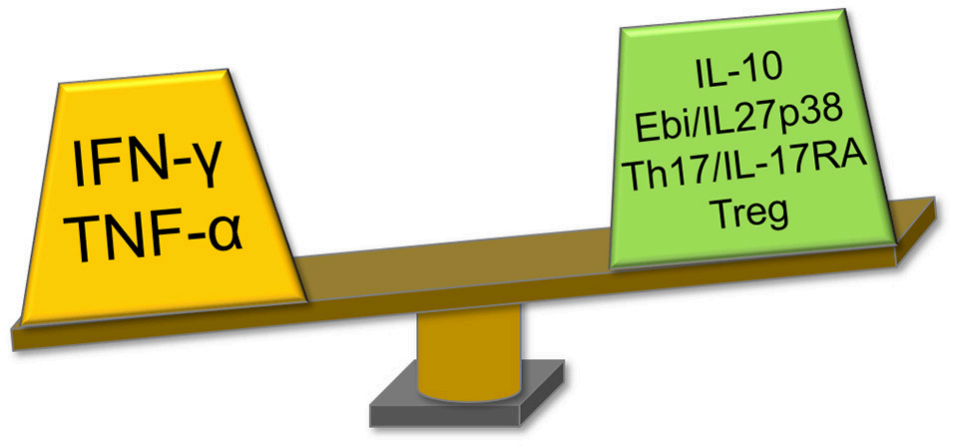
Immune profile of CCC patients revolves around the IFN-γ axis. CCC patients display increased production of PRGs IFN-γ and TNF-α, and decreased levels of IFN-γ/Th1 suppressive factors as compared with ASY patients. IL-10 and Ebi3/IL-27p28 are DTGs and IL-17, also reduced in CCC, is included here as the DTG IL17RA signaling showed to suppress the IFN-γ/Th1 axis. CCC, Chagas disease cardiomyopathy; PRG, pathogen resistance gene; ASY, asymptomatic; DTG, disease tolerance gene; IFN-γ, interferon-gamma; TNF-α, tumor necrosis factor alpha; IL, interleukin; Ebi3, Epstein-Barr virus induced gene 3; IL17RA, interleukin 17 receptor A.

Chronically infected individuals display differential disease progression. While 60% of infected individuals remain disease-free for life, 30% develop life-threatening CCC—a severe inflammatory dilated cardiomyopathy, years after infection. Ten percent develop gastrointestinal disease. CCC has a worse prognosis than cardiomyopathies of non-inflammatory etiologies, such as ischemic or idiopathic cardiomyopathy (76). CCC patients show an increased number of IFN-γ-producing Th1 T cells and plasma TNF-α levels as compared with ASY. Conversely, numbers of IL-10-producing CD4+CD25+ regulatory T cells (Tregs) CD4+CD25+ FoxP3+ Tregs and Th17 cells, as well as Ebi3/IL-27p28 levels are lower as compared with ASY (28, 31, 39, 44, 60, 77) (Figure 2). The exacerbated Th1 response observed in the peripheral blood of CCC patients is reflected on the Th1-rich inflammatory infiltrate predominantly secreting IFN-γ and TNF-α. A lower, but significant, production t of IL-4, IL-6, IL-7, IL-15, IL-18 was found in their heart tissue (57, 78–83). Indeed, IFN-γ is the most upregulated cytokine in CCC heart tissue; concordantly, we observed significant expression of T-bet, the hallmark Th1 transcription factor, in the CCC myocardium (84). We found a positive correlation between *Tbet* expression and left ventricular dilation, corroborating the pathogenic role of *Tbet* positive/IFN-γ producing T cells toward CCC. Conversely, mRNA expression of *GATA3, ROR*γ*T*, and *FoxP3*, hallmark transcription factors of Th1-antagonizing Th2, Th17, and Treg populations was low or undetectable. In agreement, mRNA expression of their signature cytokines *IL4, IL13, IL17, IL10*, and Treg molecular markers *FOXP3* and *CTLA4* was also low or undetectable (84). IFN-γ-producing CCR5+CXCR3+ Th1 T cells are more abundant in CCC than ASY (85), and the same cells were identified in CCC heart tissue, along with their chemokine ligands (CCL3, CCL4, CCL5, CXCL9, and CXCL10, respectively). *CCL5* and *CXCL9* were the most highly expressed chemokine mRNAs, and the intensity of the myocardial inflammation was positively correlated with *CXCL9* mRNA expression (86, 87). Together, this suggests that locally produced Th1 T cell-attracting chemokines play a role in the selective accumulation of Th1 T cells in CCC hearts. Moreover, it indicates that the Th1 infiltrate in CCC myocardium is essentially unopposed by regulatory cells or cytokines, suffering little regulation. This lack of regulation could explain the destructiveness of the inflammatory infiltrate, most likely due to excessive collateral damage by IFN-γ-producing T cells as described in acutely *T. cruzi*-infected mice. IFN-γ is thus considered the culprit of CCC. We believe the unopposed IFN-γ action is linked to the fact that DTGs that reduce IFN-γ production and/or Th1 T cell differentiation *IL10, Ebi3/*IL27p28, and genes involved in IL-17 signaling are downregulated in CCC patients.

The immunomodulatory profile of ASY patients, with increased levels of DTG and lower levels of the PRG IFN-γ, indicates that ASY patients are in a state of disease tolerance. It is interesting to notice that the majority of chronic Chagas disease patients −60%- are disease-tolerant ASY patients. This is in line with evolutionary studies indicating that selection of DTG is dominant over PRG, leading to higher frequencies of individuals expressing DTG than PRG.

## Deleterious effects of IFN-γ

While IFN-γ can control parasites, excessive levels can cause tissue damage and death in the acute and chronic phases. IFN-γ effectively regulates the expression of over 1,000 genes through activation of Janus tyrosine kinase (JAK) and phosphorylation of transducer and activator of transcription 1 (STAT-1) pathway, along with other mechanisms (88, 89). Among key IFN-γ-inducible inflammatory genes are TNF-α, and several other inflammatory cytokines and chemokines, interferon-inducible factor 1 (IRF1) and other PRG, including inducible nitric oxide synthase (NOS2) (90). Multiple findings suggest that IFN-γ plays a central pathogenic role in the myocarditis and heart failure in CCC patients. Systemic *T. cruzi* persistence drives continued production of IFN-γ by T cells, important for parasite control through NOS production, as well as activating ROS through induction of NADP oxidases and mitochondrial ROS through NF-kB (47, 48). However, *T. cruzi* is highly resistant to ROS; IFN-γ and the accompanying NOS and ROS can also induce severe disturbances of heart function. IFN-γ can modulate gene expression in immune cells and cardiomyocytes. A significant proportion of genes modulated in the CCC myocardium and hearts from acutely infected mice are inducible by IFN-γ (86, 91). Significantly, transgenic mice overexpressing IFN-γ develop a TNF-α-dependent myocarditis and cardiomyopathy (92, 93). In cardiomyocytes, IFN-γ treatment reduced contractility, induced NO/peroxynitrite-dependent cardiomyocyte apoptosis, reduced cardiomyocyte area, and also induced atrial natriuretic factor and production of chemokines CCL3, CCL5, and CXCL1 (94–96). IFN-γ regulates cardiac fibrosis by increasing fibroblast proliferation, production of hyaluronan and metalloproteinases 2 and 9 (95–98).

## IFN-γ and mitochondria: dangerous liaisons

Evidence of mitochondrial dysfunction has been found in hearts of animal models of Chagas disease, as well as the myocardium of CCC patients. This is especially relevant for CCC pathogenesis, since mitochondrial dysfunction is a paramount feature of heart failure of diverse etiologies (99). Nisha Garg and her group pioneered and studied in detail mitochondrial dysfunction in hearts of murine models of acute and chronic Chagas disease and *in vitro* infection models (100). Regarding mitochondrial damage in human CCC, our group described altered expression of mitochondrial genes and 16S mitochondrial rRNA in CCC heart lesions (57). We also found a selective reduction of protein expression of ATP synthase and creatine kinase activity—key mitochondrial energy metabolism enzymes- in CCC heart lesions (101). In agreement, mitochondrial DNA content was found to be reduced in CCC heart tissue (102), further indicating that mitochondrial function is compromised in CCC. Evidence indicates that many damaging effects of IFN-γ are secondary to promoting peroxynitrite-dependent and independent mitochondrial dysfunction and oxidative stress. IFN-γ effects on mitochondria include inhibition of the oxidative metabolism (103) an increased rate of ATP depletion (104) and inhibition of creatine kinase expression (105, 106). Moreover, IFN-γ+TLR ligand (LPS) treatment of cardiomyocytes– but not *T. cruzi* infection *per se*—downregulates expression of multiple mitochondrial genes. These genes were involved in the electron transport chain, mitochondrial fission, mitophagy, mitochondrial, and nuclear gene transcription (51). NF-kB activation is one of the main mechanisms of mitochondrial damage induced by IFN-γ. Although IFN-γ does not directly activate NF-kB, it enhances TNF-α-induced NF-kB nuclear translocation. IFN-γ/TNF-α-driven NF-kB activation is known to cause dissipation of the proton gradient and impairment of the mitochondrial membrane potential (MMP) and ATP synthesis, leading to apoptosis (106, 107). Persistent activation of NF-kB by IFN-γ/TNF-α has been described to potentiate ROS release, which can in turn increase tissue damage and mitochondrial energy imbalance (106). Indeed, inhibition of NF-kB has been shown to improve MMP with substantial decrease of NOS2/NO induction and ROS release. Taken together, these reports strongly suggest that IFN-γ may play a significant role in mitochondrial dysfunction and heart failure. Excessive mitochondrial ROS production by cardiomyocytes is considered as a central cause of heart failure (108, 109).

**Figure 3:**
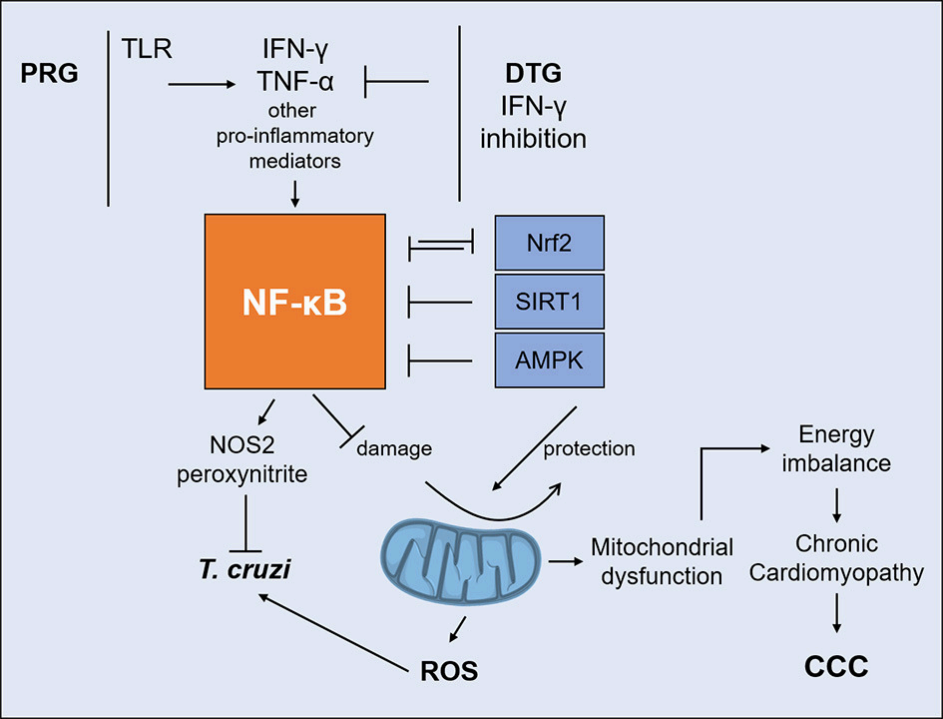
Interplay between IFN-γ and DTG determines the fate of mitochondria. IFN-γ/TNF-α strongly activate NF-kB signaling. Unchecked NF-kB activation leads to mitochondrial dysfunction, including decreased MMP, increased ROS production and reduced ATP production. IFN-γ-lowering DTG can reduce IFN-γ/TNF-α activation of NF-kB and shift the balance favoring SIRT1, Nrf2, and AMPK. Agonists can protect mitochondria and myocardial damage by reverting NF-kB activity in experimental CCC. DTG, DISEASE tolerance gene; IFN-γ, interferon-gamma; TNF-α, tumor necrosis factor alpha; NF-kB, nuclear factor kappa B; SIRT1, Sirtuin-1; Nrf2, nuclear respiratory factor 2; AMPK, 5′ adenosine monophosphate-activated protein kinase; MMP, mitochondrial membrane potential; ROS, reactive oxygen species; ATP, adenosine triphosphate.

A significant crosstalk occurs between NF-kB and mitochondrion-protecting proteins. NF-kB signaling down-regulates sirtuin-1 (SIRT1) activity through the expression of IFN-γ, ROS, and NO (110). SIRT1, an antioxidant and anti-inflammatory protein, regulates the oxidative respiration and cellular survival and is highly expressed in the heart, acting as an inhibitor of NF-kB inflammatory signals (111). While NF-kB stimulates glycolytic energy flux in acute inflammation, SIRT1 inhibits NF-kB and enhances mitochondrial oxidative metabolism through 5′ AMP-activated protein kinase (AMPK) resulting in the resolution of inflammation (110). Treatment of *T. cruzi*-infected mice with SIRT1 and/or AMPK agonists SRT1720, resveratrol and metformin reduced myocardial NF-kB transcriptional activity, inflammation and oxidative stress, resulting in beneficial results for restoration of cardiac function (100, 112). Preserving Nrf2 activity was shown to arrest the mitochondrial and cardiac oxidative stress, cardiac fibrosis, and heart failure in murine *T. cruzi* infection (113). Nrf2 is the master regulator of the antioxidant response, a transcription factor controlling expression of hundreds of genes (114), and promotes mitochondrial biogenesis (115). HMOX1 (Heme oxygenase 1), a key effector of the Nrf2 antioxidative response, is upregulated in the hearts of acutely infected mice (91), but not in the myocardium of CCC patients. Furthermore, its paralog HMOX2 is downregulated in CCC myocardium (ECN and CC, submitted for publication), indicating that Nrf2-dependent antioxidative defenses are even lower in CCC myocardium than in acute *T. cruzi* myocarditis (91). It has been recently observed that mitochondrial damage due to any stimulus leads to inflammation. Figure 3 shows the interplay between IFN-γ, DTG, NF-kB, and its antagonists, mitochondrial protective factors SIRT1, Nrf2, and AMPK vs. the outcome of mitochondrial function and inflammation.

**Figure 4:**
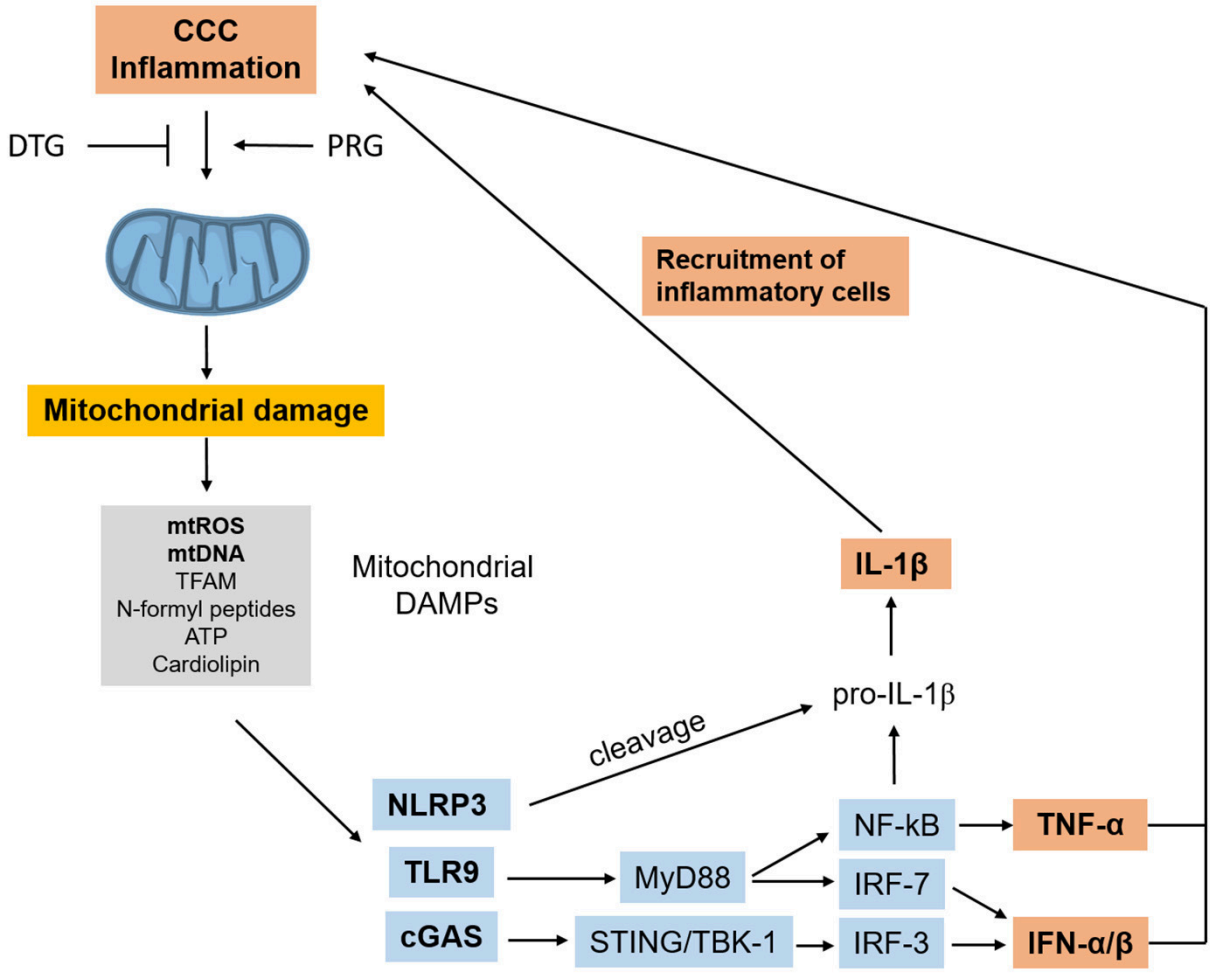
Inflammation-induced mitochondrial dysfunction starts a positive feedback loop. Inflammation in CCC can trigger mitochondrial dysfunction and damage, leading to release of mtROS and other mitochondrial DAMPs which can trigger inflammation cascades themselves, using several pathways. Such mitochondrial DAMP-induced inflammation can perpetuate inflammation and promote further mitochondrial dysfunction in a positive feedback loop. “Sterile” cardiac inflammation mediated by mitochondrial damage could thus perpetuate and/or potentiate inflammation in the hearts or CCC patients. CCC, Chagas disease cardiomyopathy; DAMPs, damage-associated molecular patterns; mtROS, mitochondrial reactive oxygen species; mtDNA, mitochondrial DNA; TFAM, transcription factor A, mitochondrial; cGAS, Cyclic GMP-AMP Synthase; NRLP3, NLR family pyrin domain containing 3; TLR9, toll-like receptor 9; MyD88, myeloid differentiation primary response 88; STING, stimulator of interferon genes protein; TBK1, TANK binding kinase 1; IRF, interferon regulatory factor; NF-kB, nuclear factor kappa B; IFN, interferon; TNF-α, tumor necrosis factor alpha.

ROS activate NF-kB, and dysfunctional or damaged mitochondria release mitochondrial DAMPs (damage-associated molecular patterns) including mitochondrial DNA (mtDNA) leading to the activation of the TLR9/Myd88/NF-kB, cGas/Type I IFN, and NRLP3 inflammasome pathways (116). In summary, the release of mitochondrial DAMPs in response to initial inflammatory stimuli and infection can establish a sterile inflammation process, a self-perpetuating positive feedback loop of mitochondrial damage and inflammation. This process has been found to occur in heart failure of diverse etiologies, and has been called sterile cardiac inflammation (117). Such sterile inflammation could be a contributing factor for the maintenance of inflammation in the absence of *T. cruzi* and may potentiate inflammatory damage in CCC (Figure 4). Together, these results indicate that mitochondrial dysfunction and mitochondrial ROS production are major pathogenic factors and therapeutic targets in CCC.

## Host genetic factors associated to differential disease progression

The finding that 30% of Chagas disease patients develop CCC suggested the participation of genetics in differential disease progression. This was reinforced by the finding of familial aggregation of cases of CCC in endemic area settings (118). CCC patients display a more intense inflammatory response than the ASY patients, who seem to have a more regulated immune response. Given the importance of inflammatory mechanisms for CCC pathogenesis, genetic susceptibility to CCC may result from functionally relevant genetic polymorphisms that lead to variations in the intensity of the innate or acquired immune response and in inflammatory cytokines and chemokines involved in the pathogenesis of the disease. Common genetic association studies compare frequencies of a genetic polymorphism (single nucleotide polymorphism, SNP) in a candidate gene (picked by the investigator) in two populations (typically disease and control). If a gene variant is more frequent in the disease group, it is said to be associated with the disease and assumed to confer risk toward developing disease. These studies show genetic contributions that are typically small, explaining < 10% of the phenotype of complex, polygenic diseases like CCC. A SNP found to be associated with a disease may be directly connected to a phenotype (e.g., a polymorphism in a transcription factor-binding sequence in the promoter region, affecting gene expression) or merely a marker of a linked biologically relevant polymorphism. In the case of Chagas disease, we performed a thorough literature search at the PUBMED database and retrieved 145 association studies addressing polymorphisms in 76 genes, which disclosed 62 SNPs from 44 genes to be associated with CCC. From those, 9 SNPs/genes were associated with CCC severity—SNPs were more frequent among severe CCC patients, with significant left ventricular dysfunction (ejection fraction < 40%), as compared to the remaining CCC patients.

In our literature search, we found that 7 genes with polymorphisms associated with CCC or severe CCC were also defined as PRG or DTG in mouse knockout infection studies (Table 2). The 5 PRG belonged to the TLR, Th1, or chemokine pathways/processes. Since TNF-α signaling is key in both the acute infection and CCC, we added *TNFA* and TNF-receptor alpha (*TNFRSF1A*) to this table, even though *TNFA* was only assessed in association studies (120–123, 132, 133, ), and *TNFRSF1A* only in knockout infection experiments (26). The two DTG Ebi3/IL27p28 and IL-10 both modulate IFN-γ via downregulation of IFN-γ production and Th1 differentiation. Such genes are central in the pathogenesis of CCC, as IFN-γ and TNF-α-producing cells migrate to CCC heart tissue in response to chemokines CCL2 and CCR5. While the−308 variant of *TNFA* has been associated with increased production, this has not been validated every time. Significantly, polymorphisms in several other genes belonging to the same pathways (*TLR-IL12-IL18-IFNG-TNFA*-NF-kB, chemokines/receptors, inflammasome including both *IL1B*, and *IL18*) have been associated with CCC.

**Table 2:** PRG and DTG gene polymorphisms associated with CCC, severe CCC, or death.

**Gene**	**Name**	**Gene type (KO studies)**	**Associated polymorphisms (patients)**	**Phenotype**	**Association study references**
**TLR PATHWAY**					
*TLR4*	Toll Like Receptor 4	PRG	D299G/T399I	CCC	(119)
**TNF/TNFR**					
*TNFA*	Tumor necrosis factor alpha		rs1799964, rs1800629, TNFα −238, *TNFα* −308	CCC	120, 121, 122
*TNFA*	Tumor necrosis factor alpha		−308, TNFα2	CCC - death	(123)
**Th1/IFN**γ					
*IL12B*	Interleukin 12A	PRG	IL12B+1188	CCC	(124)
*IFNG*	Interferon gamma	PRG	rs2430561	CCC	(125, 126)
**CHEMOKINES**					
*CCL2*	C-C Motif Chemokine Ligand 2	PRG	CCL2-2518, rs2530797, rs4586, rs3917891	CCC	(127)
*CCR5*	C-C Motif Chemokine Receptor 5	PRG	*CCR5+59029, rs1799987, rs2856758, rs2734648, rs3176763, rs11575815, rs1799988*	CCC	(87, 127–129)
*CCR5*	C-C Motif Chemokine Receptor 5	PRG	*rs1800024*	CCC - severity	(130)
**IFN**γ**-INHIBITING GENES**					
*IL10*	Interleukin 10	DTG	IL10-1082	CCC	(131)
*EBI3*	Epstein-Barr Virus Induced 3/IL-27p28	DTG	rs4740, rs4905	CCC severity	(39)

*CCC severity, associated with CCC with ventricular dysfunction (Left ventricular ejection fraction < 40%)*.

The heterozygous *TLR4*-D299G/T399I genotype occurred more frequently in ASY infected subjects than CCC patients (OR = 5.4, 95% CI = 1.03–52.6, *P* = 0.02) (119). In humans, D299G/T399I reduces TLR4 responsiveness to lipopolysaccharide and associates with increased susceptibility and/or severity of numerous infectious and inflammatory diseases. The *TIRAP* variant S180L was found to be more prevalent in the ASY group than in the CCC group (OR = 0.31, 95%CI = 0.16–0.60, *P* = 0.0084) (134). Mutation leads to a decrease in signal transduction upon ligation of TLR2 or TLR4 to their respective ligands. Weitzel et al. have shown a similar trend of association for TLR4 (119). A similar study on a large Brazilian population found that *TIRAP* rs8177376A/A is associated with an increased susceptibility to CCC (OR = 1.36; 95%CI = 1.19–1.80, *P* = 0.037) (127). This same *TIRAP* variant confers protection against malaria, tuberculosis, pneumococcal disease, and bacteraemia (135). Polymorphisms in the *TNFA-LTA* region have been extensively studied. *TNFA –LTA* polymorphisms (−308, −244, and −238) were studied on a Peruvian cohort with negative results (132), while it was found to be associated in a Mexican population (OR = 3.03, 95% CI = 1.29–7.12, *P* = 0.008) (120). Pissetti et al. have shown that the *LTA* (+252) polymorphism was significantly associated with Chagas disease as compared with healthy seronegative individuals in a Brazilian population (133). Pissetti et al. have shown that this allele is associated with higher TNF-α production in Brazilian Chagas disease patients as compared to healthy seronegative individuals (OR = 1.846, 95%CI = 1.057–3.223, *P* = 0.03) (121). Similar results were detected on a Colombian cohort (122). Severe CCC patients with significant ventricular dysfunction carrying the susceptibility genotypes *TNFA*−308 or *TNFA* A2 microsatellite display a significantly shorter survival time than those carrying other alleles (OR = 0.51, 95%CI = 0.27–0.97, *P* = 0.04) (123). CCL2 is an important chemokine attracting monocytes/macrophages to inflamed tissue. The plasma concentrations CCL2 are associated with myocardial dysfunction in patients with severe CCC, dilated cardiomyopathy and acute myocardial infarction (55, 136, 137). Ramasawmy et al. have shown on their Brazilian population that the variant at position *CCL2*−2518 A allele conferred susceptibility to CCC in a Brazilian population (OR = 4.1; 95%CI = 1.7–11; *P* = 0.001) (138), a result that was subsequently confirmed (rs2530797: OR = 1.46; 95%CI = 1.11–1.92, *P* = 0.007) (127). Multiple studies have identified associations between different polymorphisms in the *CCR5* gene (87, 127–129, 139–142). A polymorphism in *IFNG* rs2430561 was found to be associated with CCC (126). The *IL12B* +1188 C allele was found to be associated with CCC in a similar population (OR = 3.39, 95%CI = 1.3–9.15, *P* = 0.015) (124).

Finally, genes tagged as DTG were also associated in genetic studies. The functional IL10 gene polymorphism −1082G/A is associated with the development of CCC in a Brazilian population (OR = 0.84, 95%CI = 1.44–4.95, *p* < 0.01) (131). Similar data were obtained in Colombia (143). Ebi3/IL-27p28 regulates IFN-γ -mediated myocarditis by promoting an anti-inflammatory environment through IL-10. Medina et al. has shown that *Ebi3* polymorphism (SNP rs4740) is less frequent in patients with severe cardiopathy (6.6%) compared to patients with the indeterminate form (OR 3.157, 95% CI = 1.15–8.64, *p* = 0.025) (39).

## Does *T. cruzi* genetic variability play a role in determining the outcome of human chagas disease?

In addition to host genetic susceptibility factors, genetic variation in *T. cruzi* may also play a role in the outcome of Chagas disease. In animal models of infection, different *T. cruzi* strains show very distinct patterns of acute phase death, induction/activation of cytokines, PRGs and DTGs, tissue tropism, and development of chronic infection (144, 145).

Major subgroups of *T. cruzi* genetic variability have been classified into 6 discrete type units (DTU)- TcI to TcVI, each with a distinct geographical distribution. It has been reported that TcI (Colombian) and TcII (Y) strains differentially activate human monocytes (146). However, there was no difference in the DTUs found in the CCC and the ASY groups in a same geographical region; heart disease is caused by the DTUs that are prevalent in a given region (147–150) Although DTUs were not clearly associated with Chagas disease outcomes, *T. cruzi* genetic complexity surpasses the simple DTU classification. It is likely that yet undisclosed genetic variants of *T. cruzi* may influence the development of CCC, as shown in mice (145), which will certainly interact with the host genome to lead to different outcomes. Infection of BALB/c or C57BL/6 mice leads to differential activation of PRGs (151).

## Concluding remarks

The finding that all identified *T. cruzi* DTGs converge on the inhibition of IFN-γ production and/or Th1 T cell differentiation indicates the importance of this homeostatic control to avoid IFN-γ-induced death in the acute phase. Downmodulation of the IFN-γ response by DTGs favors *T. cruzi* evasion and the establishment of chronic infection. Among chronically infected patients, disease progression is associated with distinct immune profiles. In chronically infected ASY patients, IFN-γ production levels are adequately modulated by DTG *IL10, Ebi3*/IL27p28, Th17/*IL17RA* signaling, along with Treg, promoting a state of disease tolerance. Conversely, in CCC, an imbalance between DTG and *IFNG* causes increased IFN-γ production associated with inflammatory cardiac damage. Excessive myocardial IFN-γ signaling can lead to mitochondrial dysfunction. In turn this leads to energy imbalance, impairment of contractile function. Mitochondrial dysfunction induces further inflammation, in a positive feedback loop that can help perpetuate myocardial inflammation in CCC.

## Author contributions

EC-N and CC participated in conceptual design, manuscript drafting, and revision. JN and RA participated in manuscript drafting, and revision. AF, RP, and MN participated in manuscript drafting. JK participated in manuscript revision.

### Conflict of interest statement

The authors declare that the research was conducted in the absence of any commercial or financial relationships that could be construed as a potential conflict of interest.
